# Supplementary material for: Factors associated with recovery from stunting at 24 months of age among infants and young children enrolled in the Pediatric Development Clinic (PDC): A retrospective cohort study in rural Rwanda
Source: PLoS One. 2023 Jul 7;18(7):e0283504. doi: 10.1371/journal.pone.0283504 (PMC10328318; doi:10.1371/journal.pone.0283504)
Supplement: S1 Table — (DOC) [file pone.0283504.s002.doc]

**S1 Table. Comparing the characteristics of PDC children who were stunted at age 11 months by their eligibility status for inclusion in the analysis for stunting recovery at age 24 months, N=403** unless otherwise indicated

|  | **Study eligibility status at 24 months of age** | | |
| --- | --- | --- | --- |
|  | **Not eligible**  **(N=224)** | **Eligible**  **(N=179)** | **p-value^b^** |
|  | **n (%)** | **n (%)** |  |
| **District** |  |  | 0.067 |
| Kirehe | 78 (34.8) | 47 (26.3) |  |
| Kayonza | 146 (65.2) | 132 (73.7) |  |
| **PDC site** |  |  | 0.021 |
| Hospital PDCs | 101 (45.1) | 102 (57.0) |  |
| Health Center PDCs | 123 (54.9) | 77 (43.0) |  |
| **Child’s sex** |  |  | 0.759 |
| Male | 132 (58.9) | 109 (60.9) |  |
| Female | 92 (41.1) | 70 (39.1) |  |
| **Having health insurance, N=349** |  |  | 0.102 |
| No | 29 (15.0) | 14 (9.0) |  |
| Yes | 164 (85.0) | 142 (91.0) |  |
| **Age of the mother at child's enrollment to PDC [N=379] (median, IQR)** | 27 (22, 23) | 28 (23, 34) | 0.309 |
| **Mother's marital status, N=365** |  |  | 0.212 |
| Married | 134 (67.0) | 97 (58.8) |  |
| Cohabitating (Living with partner) | 44 (22.0) | 49 (29.7) |  |
| Single, divorced, or widowed | 22 (11.0) | 19 (11.5) |  |
| **Mother’s number of years in school [N=345] (median, IQR)** | 5 (3, 6) | 5 (3, 6) | 0.339 |
| **Household Socioeconomic Category (Ubudehe), N=338** |  |  | 0.658 |
| Category 1 (very poor) | 16 (8.8) | 15 (9.6) |  |
| Category 2 (poor) | 106 (58.2) | 97 (62.2) |  |
| Category 3 or 4 (not poor) | 60 (33.0) | 44 (28.2) |  |
| **Total number of children in the household** |  |  | 0.496 |
| < 3 children | 85 (38.0) | 70 (39.1) |  |
| ≥ 3 children | 105 (46.9) | 75 (41.9) |  |
| Missing data | 34 (15.2) | 34 (19.0) |  |
| **Gestational age, N=292** |  |  | >0.999 |
| ≥37 weeks | 59 (35.8) | 46 (36.2) |  |
| <37 weeks | 106 (64.2) | 81 (63.8) |  |
| **Small for gestational age, N=264** |  |  | 0.257 |
| No | 63 (43.4) | 43 (36.1) |  |
| Yes | 82 (56.6) | 76 (63.9) |  |
| **Child’s weight at birth, N=360** |  |  | 0.813 |
| >2500g | 37 (18.9) | 31 (18.9) |  |
| 2000-2499g | 36 (18.4) | 24 (14.6) |  |
| 1500-1999g | 80 (40.8) | 72 (43.9) |  |
| <1500g | 43 (21.9) | 37 (22.6) |  |
| **Child diagnosed as preterm or LBW, N=398** |  |  | 0.902 |
| No | 49 (22.1) | 37 (21.0) |  |
| Yes | 173 (77.9) | 139 (79.0) |  |
| **Child diagnosed with HIE, N=398** |  |  | 0.256 |
| No | 183 (82.4) | 137 (77.8) |  |
| Yes | 39 (17.6) | 39 (22.2) |  |
| **Child diagnosed with other conditions^a^, N=398** |  |  | 0.638 |
| No | 210 (94.6) | 169 (96.0) |  |
| Yes | 12 (5.4) | 7 (4.0) |  |
| **Child diagnosed with multiple conditions, N=398** |  |  | 0.088 |
| No | 218 (98.2) | 167 (94.9) |  |
| Yes | 4 (1.8) | 9 (5.1) |  |
| **Wasting status at closest visit to 6 months, N=394** |  |  | 0.727 |
| Not wasted (Normal WLZ) | 167 (76.3) | 137 (78.3) |  |
| Moderate Wasting | 32 (14.6) | 26 (14.9) |  |
| Severe wasting | 20 (9.1) | 12 (6.9) |  |
| **Underweight status at closest visit to 6 months, N=396** |  |  | 0.436 |
| No underweight (Normal WAZ) | 60 (27.4) | 57 (32.2) |  |
| Moderate Underweight | 80 (36.5) | 55 (31.1) |  |
| Severe Underweight | 79 (36.1) | 65 (36.7) |  |
| **Stunting status at closest visit to 6 months, N=392** |  |  | 0.825 |
| Not stunted (Normal LAZ) | 55 (25.2) | 42 (24.1) |  |
| Moderate Stunting | 82 (37.6) | 71 (40.8) |  |
| Severe Stunting | 81 (37.2) | 61 (35.1) |  |
| **Wasting status at closest visit to 11 months, N=398** |  |  | 0.214 |
| Not wasted (Normal WLZ) | 176 (78.9) | 134 (76.6) |  |
| Moderate Wasting | 29 (13.0) | 32 (18.3) |  |
| Severe wasting | 18 (8.1) | 9 (5.1) |  |
| **Underweight status at closest visit to 11 months, N=402** |  |  | 0.688 |
| No underweight (Normal WAZ) | 77 (34.5) | 55 (30.7) |  |
| Moderate Underweight | 71 (31.8) | 58 (32.4) |  |
| Severe Underweight | 75 (33.6) | 66 (36.9) |  |
| **Stunting status at closest visit to 11 months** |  |  | 0.368 |
| Moderate Stunting | 109 (48.7) | 79 (44.1) |  |
| Severe Stunting | 115 (51.3) | 100 (55.9) |  |
| **History of feeding difficulties, N=297** |  |  | 0.157 |
| No | 130 (75.1) | 102 (82.3) |  |
| Yes | 43 (24.9) | 22 (17.7) |  |
| **^a^**Other conditions include the following: central nervous system infections, trisomy 21, post-hospitalization for severe malnutrition when < 12 months of age, hydrocephalus, cleft lip or palate and other developmental delays.  **^b^**Fisher’s exact test was used for categorical variables and Wilcoxon Rank Sum test was used for continuous variables  **Abbreviations:** HIE, hypoxic ischemic encephalopathy; IQR, interquartile range; LBW, low birth weight; LAZ, length-for-age z-scores; WLZ, weight-for-length z-scores; WAZ, weight-for-age z-scores | | | |
