# Supplementary material for: Factors associated with recovery from stunting at 24 months of age among infants and young children enrolled in the Pediatric Development Clinic (PDC): A retrospective cohort study in rural Rwanda
Source: PLoS One. 2023 Jul 7;18(7):e0283504. doi: 10.1371/journal.pone.0283504 (PMC10328318; doi:10.1371/journal.pone.0283504)
Supplement: S2 Table — (DOC) [file pone.0283504.s003.doc]

**S2 Table**. Bivariate analysis of the factors associated with stunting recovery at 24 months of age among PDC children identified with stunting at age of 11 months, N=179 unless otherwise indicated

|  | **Stunting recovery at 24 months of age** | | | | |
| --- | --- | --- | --- | --- | --- |
|  | **No recovery**  **(n=142, 79.3%)** | | **Recovered**  **(n=37, 20.7%)** | | **p-value^b^** |
|  | **n** | **%** | n | % |  |
| **District** |  |  |  |  | >0.999 |
| Kirehe | 37 | 78.7 | 10 | 21.3 |  |
| Kayonza | 105 | 79.6 | 27 | 20.4 |  |
| **PDC site** |  |  |  |  | 0.852 |
| Hospital PDCs | 80 | 78.4 | 22 | 21.6 |  |
| Health Center PDCs | 62 | 80.5 | 15 | 19.5 |  |
| **Child’s sex** |  |  |  |  | 0.350 |
| Male | 89 | 81.7 | 20 | 18.3 |  |
| Female | 53 | 75.7 | 17 | 24.3 |  |
| **Having health insurance, N=156** |  |  |  |  | **0.075** |
| No | 14 | 100.0 | 0 | 0.0 |  |
| Yes | 110 | 77.5 | 32 | 22.5 |  |
| **Age of the mother at child's enrollment to PDC (median, IQR)** | 27 | (22, 34) | 30 | (26, 35) | **0.070** |
| **Mother's marital status, N=165** |  |  |  |  | 0.765 |
| Married | 78 | 80.4 | 19 | 19.6 |  |
| Cohabitating (Living with partner) | 40 | 81.6 | 9 | 18.4 |  |
| Single, divorced, or widowed | 17 | 89.5 | 2 | 10.5 |  |
| **Mother’s number of years in school (median, IQR)** | 5 | (3-6 ) | 4 | (3, 6) | 0.777 |
| **Household Socioeconomic Category (Ubudehe)** |  |  |  |  | 0.460 |
| Category 1 (very poor) | 14 | 93.3 | 1 | 6.7 |  |
| Category 2 (poor) | 77 | 79.4 | 20 | 20.6 |  |
| Category 3 or 4 (not poor) | 34 | 77.3 | 10 | 22.7 |  |
| Unknown | 17 | 73.9 | 6 | 26.1 |  |
| **Total number of children in the household** |  |  |  |  | 0.786 |
| < 3 children | 57 | 81.4 | 13 | 18.6 |  |
| ≥ 3 children | 59 | 78.7 | 16 | 21.3 |  |
| Missing data | 26 | 76.5 | 8 | 23.5 |  |
| **Gestational age** |  |  |  |  | 0.685 |
| ≥37 weeks | 37 | 80.4 | 9 | 19.6 |  |
| <37 weeks | 62 | 76.5 | 19 | 23.5 |  |
| Missing data | 43 | 82.7 | 9 | 17.3 |  |
| **Small for gestational age** |  |  |  |  | 0.277 |
| No | 31 | 72.1 | 12 | 27.9 |  |
| Yes | 60 | 79.0 | 16 | 21.1 |  |
| Unknown GA/Birthweight | 51 | 85.0 | 9 | 15.0 |  |
| **Child’s weight at birth, N=164** |  |  |  |  | 0.918 |
| >2500g | 24 | 77.4 | 7 | 22.6 |  |
| 2000-2499g | 19 | 79.2 | 5 | 20.8 |  |
| 1500-1999g | 57 | 79.2 | 15 | 20.8 |  |
| <1500g | 31 | 83.8 | 6 | 16.2 |  |
| **Child diagnosed as preterm or LBW, N=176** |  |  |  |  | >0.999 |
| No | 30 | 81.1 | 7 | 18.9 |  |
| Yes | 110 | 79.1 | 29 | 20.9 |  |
| **Child diagnosed with HIE, N=176** |  |  |  |  | 0.260 |
| No | 106 | 77.4 | 31 | 22.6 |  |
| Yes | 34 | 87.2 | 5 | 12.8 |  |
| **Child diagnosed with other conditions^a^, N=176** |  |  |  |  | 0.152 |
| No | 136 | 80.5 | 33 | 19.5 |  |
| Yes | 4 | 57.1 | 3 | 42.9 |  |
| **Child diagnosed with multiple conditions, N=176** |  |  |  |  | 0.688 |
| No | 132 | 79.0 | 35 | 21.0 |  |
| Yes | 8 | 88.9 | 1 | 11.1 |  |
| **Wasting status at closest visit to 6 months, N=175** |  |  |  |  | >0.999 |
| Not wasted (Normal WLZ) | 107 | 78.1 | 30 | 21.9 |  |
| Moderate Wasting | 21 | 80.8 | 5 | 19.2 |  |
| Severe wasting | 10 | 83.3 | 2 | 16.7 |  |
| **Underweight status at closest visit to 6 months, N=177** |  |  |  |  | **<0.001** |
| No underweight (Normal WAZ) | 37 | 64.9 | 20 | 35.1 |  |
| Moderate Underweight | 42 | 76.4 | 13 | 23.6 |  |
| Severe Underweight | 61 | 93.9 | 4 | 6.1 |  |
| **Stunting status at closest visit to 6 months, N=174** |  |  |  |  | **<0.001** |
| Not stunted (Normal LAZ) | 24 | 57.1 | 18 | 42.9 |  |
| Moderate Stunting | 58 | 81.7 | 13 | 18.3 |  |
| Severe Stunting | 56 | 91.8 | 5 | 8.2 |  |
| **Wasting status at closest visit to 11 months, N=175** |  |  |  |  | 0.780 |
| Not wasted (Normal WLZ) | 107 | 79.9 | 27 | 20.1 |  |
| Moderate Wasting | 24 | 75.0 | 8 | 25.0 |  |
| Severe wasting | 7 | 77.8 | 2 | 22.2 |  |
| **Underweight status at closest visit to 11 months** |  |  |  |  | **0.012** |
| No underweight (Normal WAZ) | 37 | 67.3 | 18 | 32.7 |  |
| Moderate Underweight | 46 | 79.3 | 12 | 20.7 |  |
| Severe Underweight | 59 | 89.4 | 7 | 10.6 |  |
| **Stunting status at closest visit to 11 months** |  |  |  |  | **<0.001** |
| Moderate Stunting | 50 | 63.3 | 29 | 36.7 |  |
| Severe Stunting | 92 | 92.0 | 8 | 8.0 |  |
| **History of feeding difficulties, N=124** |  |  |  |  | >0.999 |
| No | 87 | 84.5 | 16 | 15.5 |  |
| Yes | 18 | 85.7 | 3 | 14.3 |  |
| ^a^Other conditions include the following: central nervous system infections, trisomy 21, post-hospitalization for severe malnutrition when < 12 months of age, hydrocephalus, cleft lip or palate and other developmental delays.  ^b^Fisher’s exact test was used for categorical variables and Wilcoxon Rank Sum test was used for continuous variables  Abbreviations: HIE, hypoxic ischemic encephalopathy; IQR, interquartile range; LBW, low birth weight; LAZ, length-for-age z-scores; WLZ, weight-for-length z-scores; WAZ, weight-for-age z-scores | | | | | |
